# Case Report: Food Protein-Induced Protein Losing Enteropathy (FPIPLE) in Infancy

**DOI:** 10.3389/fnut.2022.810409

**Published:** 2022-01-31

**Authors:** Gavriela Feketea, Alina Popp, Daniela Marcela Ionescu, Elena Camelia Berghea

**Affiliations:** ^1^Ph.D. School, Iuliu Hatieganu University of Medicine and Pharmacy, Cluj-Napoca, Romania; ^2^Department of Pediatrics, Pediatric Allergy Outpatient Clinic, Karamandaneio Children Hospital, Patras, Greece; ^3^Department of Pediatrics, Carol Davila University of Medicine and Pharmacy, Bucharest, Romania; ^4^National Institute for Mother and Child Health, Carol Davila University of Medicine and Pharmacy, Bucharest, Romania; ^5^Department of Pediatrics, Marie Curie Clinical Emergency Hospital for Children, Bucharest, Romania

**Keywords:** food-protein induced protein-losing enteropathy, diet, infancy, edema, hypoproteinemia, hypoalbuminemia

## Abstract

Food-protein induced protein-losing enteropathy (FPIPLE) is a mixed IgE and non-IgE food allergy in infants along with eosinophilic gastrointestinal (GI) diseases (EGID). It is characterized by poor weight gain, edema, due to hypoproteinemia/hypoalbuminemia by enteral loss of proteins, anemia, eosinophilia, raised fecal α1-antitrypsin (α1AT), and specific-IgE and allergy skin prick test (SPT) positive for offending foods. Here, we describe 4 cases with the same clinical pattern (edema due to hypoproteinemia/hypoalbuminemia from enteral loss of proteins, confirmed by high α1AT in the stools and no other pathological findings explaining the hypoproteinemia including normal kidney and liver function parameters), and propose the term “food-protein induced protein-losing enteropathy” (FPIPLE) to define this clinical entity. We also propose diagnostic criteria and an empirical algorithm of a practical approach to the diagnosis and management for children suspected to have FPIPLE. These infants can be managed successfully with dietary modification. In our 4 cases, initially, an empirical elimination diet was applied, comprising the foods that had benn introduced in the infant's diet during the last month and, an extensively hydrolyzed or elemental formula was given. In a second approach, after evaluation by a pediatric allergist, an allergy test-directed dietary elimination alimentation was implemented, for mother and/or infant. It has yet to be demonstrated whether patients with FPIPLE are a subset of patients with EGID, and whether early intervention modifies the natural course.

## Introduction

Food allergy is “an adverse health effect arising from a specific immune response that occurs reproducibly on exposure to a given food” (1). The offending foods and natural course vary with age, with implications for diagnosis and management (2). Food allergies include IgE-, non-IgE-and mixed IgE and non-IgE -mediated reactions, with diverse clinical presentations (3). In the first years of life, the non-IgE-mediated gastrointestinal (GI) food allergic diseases (non-IgE-GI-FA) including food protein-induced enterocolitis syndrome (FPIES), food protein-induced enteropathy (FPE) and food protein-induced allergic proctocolitis (FPIAP), represent the majority of food allergies (4). Combined (mixed) IgE and cell mediated (delayed onset/chronic) include atopic dermatitis (AD), eosinophilic GI diseases (EGID) eosinophilic esophagitis (EoE), eosinophilic gastroenteritis (EGE) and eosinophilic proctocolitis (5). The eosinophilic GI diseases (EGID) are described in older children, mostly due to delayed access to early endoscopy (6). Protein-losing enteropathy (PLE) is characterized by the loss of proteins through the gastrointestinal (GI) tract, leading to hypoalbuminemia and hypoproteinemia (7). PLE associated with GI allergy was described in adults more than 50 years ago (8), but data on clear diagnostic criteria in infancy are limited (9), and there is no common term to define it. Several published case reports describe infants from various populations around the world, with or without other allergic comorbidities (10–16). In this study we put together our latest cases with the same clinical pattern, namely edema due to hypoproteinemia/hypoalbuminemia from enteral loss of proteins, confirmed by high values of α1 antitrypsin (α1AT) in the stools and no other pathology explaining the hypoproteinemia including normal kidney and liver function parameters, and observed them further, with the aim of establishing a distinct clinical entity.

### Clinical Presentation and Diagnostic Assessment of “Food-Protein Induced Protein-Losing Enteropathy” (FPIPLE) in 4 Infants

Patient 1, a male, was born at full term, by vaginal delivery, after a normal pregnancy, and was exclusively breastfed. At the age of 4 months, the infant presented difficulties in breastfeeding, developed mild atopic dermatitis (AD), and his weight gain gradually fell below the expected age limits, with no other accompanying symptoms (normal stools, no vomiting, no fever). The first tests performed at the age of 5 months were completely normal: normal peripheral blood eosinophil count, normal total IgE, normal total serum protein, stools negative for occult bleeding, and negative urine and stool cultures. He continued to be breastfed and was monitored monthly for weight gain by the pediatrician. After the age of 6 months and 3 weeks, solid foods were gradually introduced in his diet (broccoli, zucchini, carrots, parsnips, sweet potatoes, chicken), but they were accepted with difficulty and only in small quantities. At the age of 7 months, yogurt and egg were offered and accepted in small quantities, and apparently well tolerated. Unsatisfactory weight gain and a capricious appetite were the reasons for referral of the child by the pediatrician to a pediatric gastroenterologist. On examination, edema was observed, and the laboratory analysis showed no proteinuria, but slightly low total serum protein level, increased total IgE, with specific IgE positive for egg white and negative for milk proteins. Based on these results, egg was excluded from the diet of both the mother and the baby. At 10 months, despite a satisfactory weight gain for his age compared with the previous month, the infant still had peripheral edema, and in addition, puffy eyes, and the laboratory tests revealed further hypoproteinemia. Stool samples showed positive occult hemorrhage and raised α1AT. The patient was diagnosed with hypoproteinemia and hypoalbuminemia from protein loss via the GI tract. Human albumin was administered intravenously (IV) twice weekly for several weeks. Despite albumin administration and disappearance of the edema, the infant's weight gain was still suboptimal, and the first allergy assessment was conducted at the age of 11 months, after other causes of hypoproteinemia had been ruled out. The relevant data from laboratory tests were eosinophilia, high levels of total IgE and specific IgE to egg white, egg yolk, ovomucoid, ovalbumin, wheat, rice, hazelnuts and peanuts but not to cow's milk protein (Table 1). Esophago-gastro-duodenoscopy and gastric, duodenal and proximal jejunal biopsy revealed mild atrophy, rare eosinophils, no celiac disease features and no lymphangiectasia. All foods for which specific modified IgE was identified were excluded from both the mother's and the baby's diet. Because of the high frequency of involvement of cow's milk proteins in inducing non-IgE-mediated digestive reactions in infants, dairy products were also excluded, and supplementation of the child's diet with aminoacid formula was prescribed. Even after the first month of diet exclusion, the clinical evolution was favorable; the edema and heme-positive stools resolved, and the serum albumin level was maintained within the normal range. After 4 months of the diet, solid foods (potato, rice, wheat) were subsequently reintroduced, with one new food per week, and they were well tolerated, with no blood loss in the stool and no peripheral edema. The reintroduction of cow's milk proteins in the diet was unsuccessfully attempted after 6 and 12 months of exclusion; raised fecal α1AT values were observed after 2–4 weeks of consumption of the offending food. After 2 years of age, the reintroduction of cow's milk proteins was again attempted, and the long-term monitoring of clinical symptoms and of fecal α1AT (at 1, 3 and 6 months after reintroduction) showed that the food allergens were very well tolerated after a longer period of exclusion. Step by step, egg proteins, hazelnuts and peanuts were introduced and tolerated. The boy is now growing well, without chronic GI symptoms.

**Table 1:** Clinical and laboratory characteristics of infants with food-protein induced protein-losing enteropathy (FPIPLE).

**Characteristics**		**Patient 1**	**Patient 2**	**Patient 3**	**Patient 4**
Sex		M	M	M	F
Age at onset		4 months	8 months	9 months	23 days
Age at diagnosis		8 months	11 months	11 months	25 days
Feeding		Breastfed	Breastfed	Breastfed	Formula
Growth failure/poor weight gain for age/dropping > 2 percentiles in WHO chart.		Yes	No	Yes	Yes
Gastrointestinal symptoms		Occult rectal bleeding no vomiting	No rectal bleeding, no vomiting	Occult rectal bleeding no vomiting	No rectal bleeding, no vomiting
Edema		Swelling of hands, foots and face	Swelling of hands and foots.	Swelling of hands and foots.	Mild swelling of hands
Atopic dermatitis		Yes	Yes	Yes	No
Family history of atopy		Allergic rhinitis	Allergic rhinitis, asthma	No	Food allergy
Laboratory data on admission	Normal values	3.62	3.7	3.4	5.3
Total protein, g/dL	5.3–7.2	2.1	2.1	2.3	2.9
Albumin, g/dL	2.9–5.5	60.12	32.16	35	43
IgE, U/mL	<15	13,100	10,480	7,730	10,400
White blood cells, per L	6–17,5	9,000	6,520	5,310	1,700
Lymphocytes, per L	2,5–10	1,660	1,580	1,140	1,600
Eosinophils, per L	<500	10.8	7.6	10.8	10.3
Hemoglobin g/dL	10.5–13.5	21	13	11	ND
Plasma iron mcg/dL	55–150	23.9	6.1	36	ND
Ferritin ng/mL	36–100	8.95	10.4	9	ND
Serum calcium mg/dL	8.0–10.7	1,800	1,414	1,800	ND
Stool α1-antitripsin μg/g	<400	Normal	Normal	Normal	Normal
Urinalysis					
Specific IgE (kU/L)	<0.35	0.1	0.32	0.61	0.36
Cow milk proteins	kU/L	0.1	0.1	0.16	2.21
Alpha-lactalbumin		0.1	0.1	11.45	0.99
Beta-lactoglobulin		0.1	0.4	0.81	0.1
Casein		1.88	0.28	0.41	ND
Egg white		0.17	0.1	0.39	ND
Egg yolk		0.2	2.2	0.1	ND
Ovomucoid		0.41	1.1	0.3	ND
Ovalbumin		0.48	0.23	0.1	ND
Wheat		0.1	0.1	3.15	ND
Soy		0.23	0.1	2.32	ND
Hazelnuts		0.23	0.1	0.29	ND
Peanuts		0.1	0.1	0.1	ND
Fish (cod)					
Biopsy (gastric, duodenal and proximal jejunal biopsy)		Mild atrophy, rare eosinophils, no celiac disease features, no lymphangiectasia	ND	ND	ND
Offending food(s)		Cow's milk, egg, potato	Cow's milk, egg	Cow's milk,	Cow's milk
				egg, soy, nuts, fish	
Human albumin i.v.		2 times	2 times	1 time	No
Management		elimination of offending foods and amino-acid formula	elimination of offending foods and amino-acid formula	elimination of offending foods and extensively hydrolyzed formula	amino-acid formula

*ND: not done*.

Patient 2, a male, was born at full term, after a normal, uncomplicated pregnancy, by vaginal delivery, and was exclusively breastfed until the age of 7 months, when solid foods were introduced in his diet. He developed persistent mild AD at the age of 2 months and edema at the age of 8 months. Similar to patient 1, he had hypoproteinemia with enteral protein loss (α1AT = 1.414 μg/g) and no other identifiable cause of hypoproteinemia. Human albumin was administered twice. Allergic investigations showed eosinophilia in the peripheral blood and sensitization to cow's milk and egg proteins. Based on these findings, he was started on an elimination diet, with avoidance of all dairy and egg products, and aminoacid formula was prescribed. Following this regimen, there was good clinical evolution, the edema disappeared and the fecal α1AT level normalized. At the age of 3 years, when skin prick test (SPT) reaction and serum IgE level had decreased, cow's milk was introduced in the diet, after a negative oral food challenge (OFC). Two months later, AD reappeared and the α1AT increased, and consequently, the consumption of cow's milk was again discontinued, but successfully reintroduced after another few months of exclusion. After 6 months of consumption of cow's milk proteins without symptoms, egg was also reintroduced in the diet and was tolerated.

Patient 3, a male, was born at full term after a normal, uncomplicated pregnancy, by cesarean section. He was breastfed for one month, then bottle-fed, and the diet was diversified at the age 6 months. He developed a transient eczema around the age of 2 months. At the age of 9 months, he was hospitalized for investigation of significant edema of the eyelids and limbs, which had developed insidiously. The laboratory tests revealed anemia, iron deficiency, eosinophilia, slightly elevated serum level of total IgE and severe hypoproteinemia, with enteral protein loss (α1AT = 1,800 μg/g) requiring human albumin administration (Table 1). An extensively hydrolyzed formula was prescribed empirically, and egg and fish, which had been introduced the previous month, were eliminated from his diet. Allergy evaluation revealed sensitivity to cow's milk protein, egg, soy and peanuts, with negative tests for fish protein. On a diet excluding all the foods with positive tests, with iron treatment for his anemia, the clinical evolution was very good, with correction of the anemia and hypoproteinemia. Fecal α1AT levels normalized after 4–6 weeks of elimination diet, and fish was reintroduced. Reintroduction of cow's milk protein was successfully started at 2 years of age, and after about 6 months of regular milk consumption, egg proteins were also reintroduced, with good clinical tolerance and without impairment of biological parameters (α1AT).

Patient 4, a female, was born at full term after a normal, uncomplicated pregnancy, by vaginal delivery, and was bottle-fed with cow's milk formula from birth. At the age of 23 days, she developed mild swelling of hands and was taken to the pediatric emergency department (ED) where she was examined by a pediatric allergist. She had no history of fever, vomiting or change in stool pattern or quality. Her examination revealed poor weight gain for age and edema over the hands, without a rash or other lesions. Laboratory tests showed hypoproteinemia, hypoalbuminemia, anemia, and eosinophilia. Urinalysis was negative for protein. SPT performed in the ED was positive for cow's milk, and further allergy evaluation showed detectable food-specific IgE antibody to cow milk proteins, α-lactalbumin and β-lactoglobulin. An aminoacid formula was prescribed, and over 2 weeks the edema resolved, and albumin level improved. She started complementary foods at 5 months and remained on aminoacid formula until the age of 7 months, when SPT and sIgE to milk were negative and an OFC to cow's milk was passed successfully.

**Figure 1:**
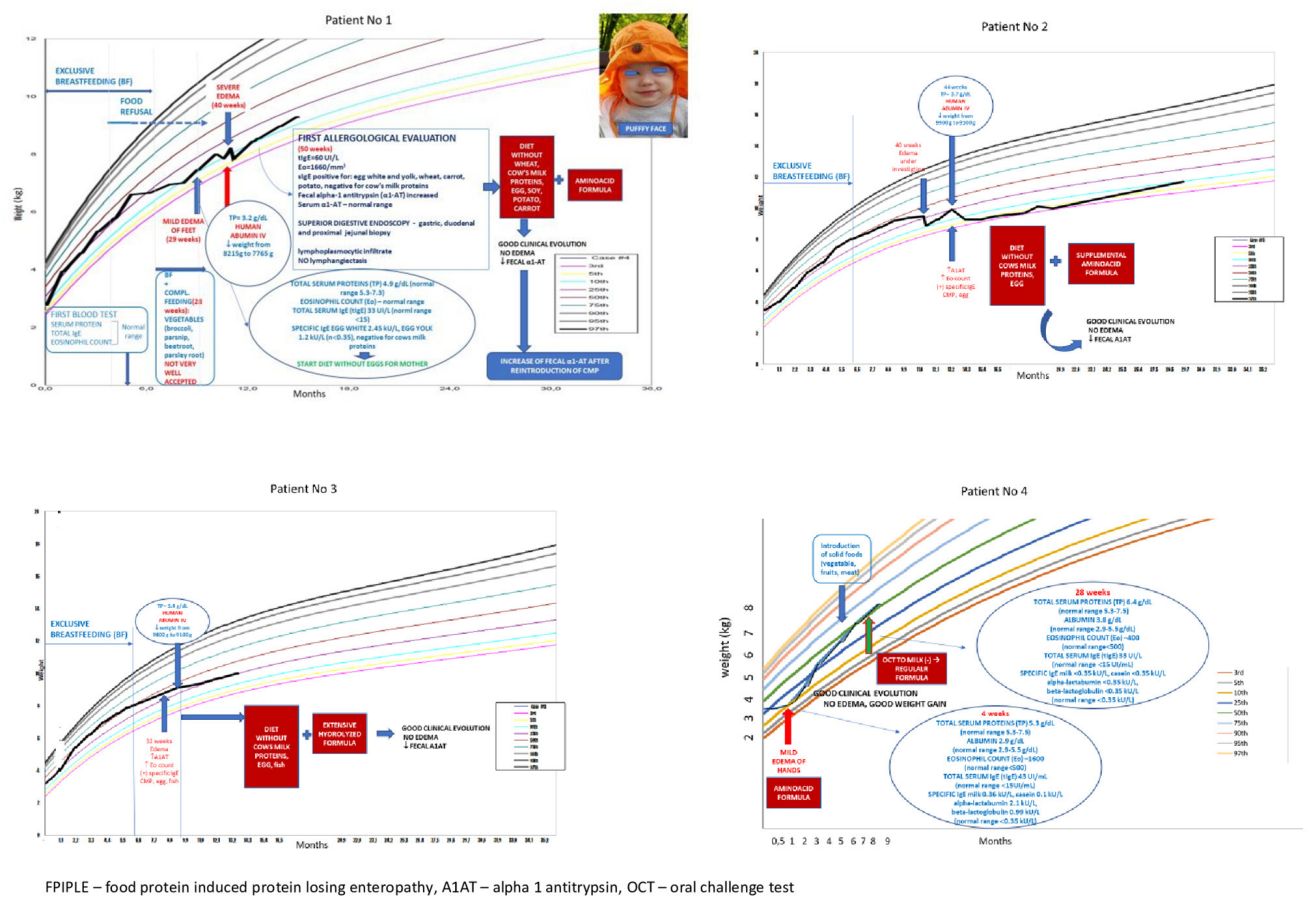
The weight charts of 4 patients with FPIPLE. OCT = oral challenge test, TP = total protein, α1-AT= fecal alpha-1 antitrypsin. FPIPLE, food-protein induced protein-losing enteropathy; A1AT, alpha 1 antitrypsin; OCT, oral challenge test.

We propose the term “food-protein induced protein-losing enteropathy” (FPIPLE) to describe this clinical entity, which would include infants with mixed IgE and non-IgE food allergy, with poor weight gain and/or edema due to hypoproteinemia/hypoalbuminemia by enteral loosing of proteins, confirmed by high values of alpha 1-antitrypsin (α1AT) in stools and no other pathology explaining the hypoproteinemia including normal kidney and liver function parameters. Additionally, they have anemia and peripheric eosinophilia. Specific IgE and allergy SPT are usually positive for the offending foods. These infants can be managed with dietary modification. Here we report 4 cases of PLE due to food allergy in infants, successfully treated by elimination of offending foods. Written consent for inclusion was obtained from their parents. The main clinical symptoms were poor weight gain and edema. With the term of “poor weight gain” we described a child or infant whose current weight, or rate of weight gain is significantly below that expected for age, sex, and ethnicity, or whose weight has dropped ≥2 major percentile lines on the WHO growth chart. Other causes of slow weight gain such as: inadequate caloric intake or excessive caloric utilization were excluded. The weight charts for all 4 patients are presented in Figure 1. None of the children had vomiting or spit-up, the frequency of bowel movements was within the normal range for age and type of feeding, and only two of the infants had occult rectal bleeding. The clinical and laboratory characteristics are summarized in Table 1. The offending foods were milk, egg, soy, potato, nuts and fish, suggesting that all the major allergenic food can trigger FPIPLE. Patient no 1 was breastfeeding exclusively when the first symptom, poor weight gain appeared. Mild edema of the feet was described for the first time, 1 week later, when still on breastfeeding, with a small amount of vegetables as complementary foods. At that time, his level of total proteins value was 4,9 g/dL (normal range 5.3–7.3) and thus the infant apparently reacted to proteins through breast milk. Because of the finding of positive IgE specific to egg, a maternal elimination diet for egg was applied and an improvement in weight gain was observed. This is in concordance with several case reports of children with PLE while on exclusively breastfeeding (12, 13, 15).

## Discussion

The diagnosis of FPIPLE was based on the clinical history, age, and laboratory findings. In patient 4, fecal α1AT was not measured at diagnosis, for technical reasons. In the first 3 patients, FPIPLE diagnosis was delayed, and administration of human albumin was necessary, but in patient 4, early diagnosis enabled prompt correction of the serum albumin level by dietary modification. In all infants, symptoms resolved with discontinuation of cow's milk formula and introduction of an amino-acid-based or extensively hydrolyzed formula, with elimination of offending foods according to IgE sensitization for patients with multiple food allergies. At the age of 3 years, when SPT and sIgE had been decreased, cow's milk was introduced in alimentation of patient no 2, after a negative OFC. Two months later, AD reappeared and α1AT increased, and consequently, the consumption of cow's milk has been again interrupted. Parallel improvement in anemia, albumin, eosinophil count and fecal α1AT level (where measured) was observed in all children.

We define FPIPLE as a clinical disorder that presents during infancy or early childhood and meets the following criteria:

- Symptoms: low weight gain for age and/or edema.
- Laboratory findings: hypoalbuminemia, hypogammaglobulinemia, IgE specific to offending food and/or certain components of offending food(s), and positive fecal α1-antitrypsine (α1AT). Anemia and eosinophilia usually are observed.
- Improvement of clinical and laboratory findings after elimination of offending food (s).

The serum protein and albumin levels reflect the balance between intake, synthesis, metabolism and loss (17). Differential diagnosis includes other clinical entities, such as low protein intake and/or production, and protein-losing syndromes (via the GI or renal tract). In adults, PLE complicates various cardiac diseases, due to increased lymphatic pressure, inflammatory intestinal disease and primary intestinal lymphangiectasia (18), all exceptionally rare in childhood.

**Figure 2:**
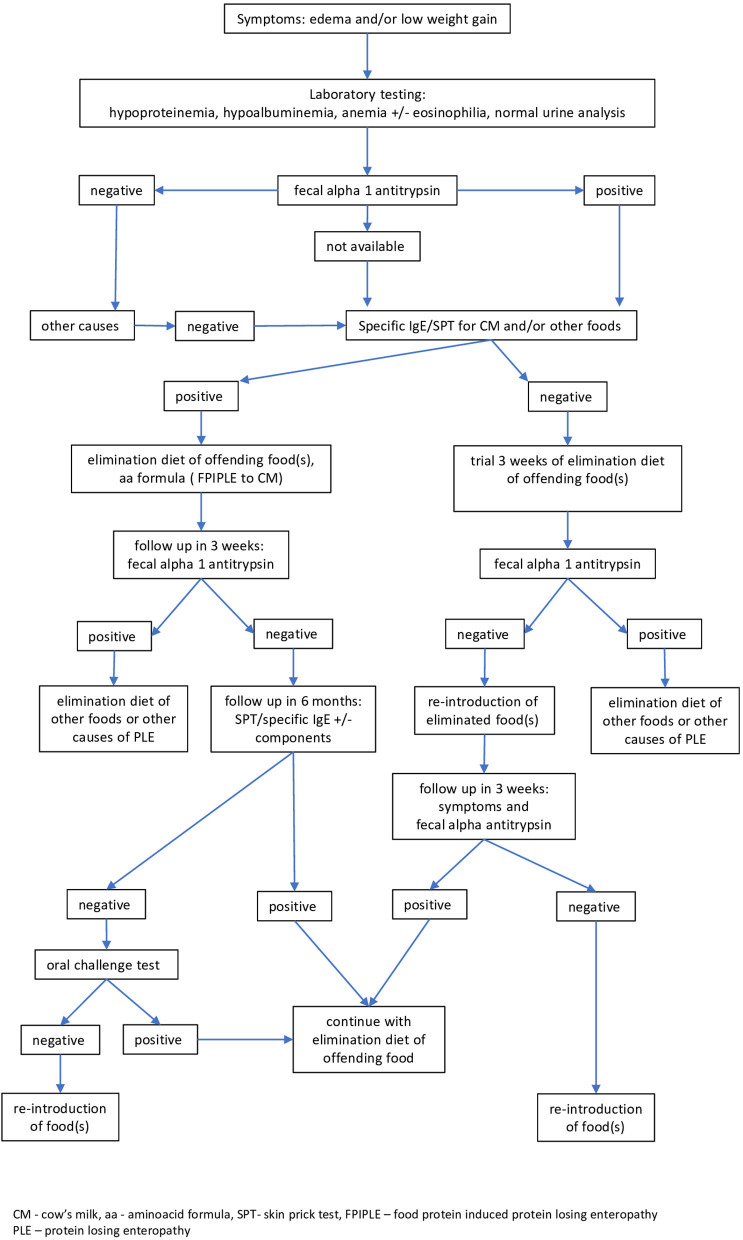
Empirical algorithm for diagnosis and management of food-protein induced protein-losing enteropathy (FPIPLE). Edema and/or low weight gain for age are suggestive of this condition. Laboratory tests should include serum total protein and albumin, hemoglobin/hematocrit, iron, IgE specific for milk and/or other incriminated foods, and fecal α1 antitrypsin. Skin testing or specific IgE tests should be repeated before an oral food challenge is considered. CM, cow's milk, aa, aminoacid formula; SPT, skin prick test; FPIPLE, food-protein induced protein-losing enteropathy; PLE, protein losing enteropathy.

Figure 2 shows an empirical algorithm of a practical approach to diagnosis and management of FPIPLE in infants. Edema and/or low weight gain for age are suggestive of this condition. Vomiting, diarrhea or rectal bleeding, symptoms common in other mixed and non-IgE mediated allergies; eosinophilic gastroenteritis (EGE), food protein-induced allergic proctocolitis (FPIAP), may be observed. Laboratory tests should include serum total protein and albumin, hemoglobin/hematocrit, iron, IgE specific for milk and/or other offending foods, and fecal α1AT for protein-losing enteropathy. Other conditions should be ruled out, including low protein intake and renal causes of hypoproteinemia and hypoalbuminemia. Skin testing or specific IgE tests should be repeated before an oral food challenge is considered. In infancy and early childhood, the main allergenic food is cow's milk, but FPIPLE can be triggered by other foods. Furthermore, based on positive specific IgE to casein, alpha-lactalbumin and beta-lactoglobulin in our cases, all proteins of cow's milk seem to induce FPIPLE.

FPIPLE is a mixed IgE and non-IgE mediated food allergy, along with eosinophilic GI diseases (EGID) which encompasses eosinophilic esophagitis (EoE), EGE and eosinophilic proctocolitis, and is clinically similar. EGID includes a heterogeneous group of diseases which have in common an eosinophilic inflammation of the gut confirmed by biopsy (19). Approximately 20% of patients with EGID present with protein-losing enteropathy (20). There are no standardized non-invasive diagnostic tests (9) and endoscopy and biopsy are the gold standard for diagnosis of EGID, but endoscopy is not always possible in early infancy. In addition, occasionally, the eosinophilic infiltration is patchy, hence the biopsy result could be inconclusive (21, 22). Infants diagnosed with FPIPLE can proceed to immediate food allergy evaluation, in parallel with exclusion of other causes of PLE, without endoscopy. Mixed food allergy (IgE and non-IgE mediated) involves low weight gain and nutritional deficiency, and immediate dietary modification is needed, in infant feeds, and/or maternal diet (23). There may be overlap with EGID (24), but the need for immediate intervention to influence the natural course, and to improve the signs and symptoms, is paramount. In both EoE and EGE cases, the patients have a history of allergic diseases and food allergy is frequently observed (25).

We believe that FPIPLE is member of allergic march and could be associated with the subsequent development of EoE, a late manifestation of the allergic march in some individuals (26). It can be speculated that the early reversal of local inflammation could prevent further sensitization to other foods. Specific proteins are the most likely culprit in this GI inflammation, but specific IgE may be positive or negative. It has yet to be demonstrated whether patients with FPIPLE are a subset of patients with EGID, and whether early intervention modifies the natural course. Nevertheless, we consider that these infants should be given the opportunity of a rapid and less expensive management through dietary intervention rather than being delayed in order to perform an invasive investigation for a histopathological diagnosis. Additionally, early treatment of FPIPLE would impede the ensuing atopic march.

These conditions are probably underrecognized in daily clinical practice and vary from simple to complex, and from mono- to multiple-food sensitization. The age appears to play a role, and it develops in both breastfed and formula-fed infants. Larger, prospective studies are needed to confirm these findings.

### Patient Perspective

It is unknown how common FPIPLE is in primary care and pediatric allergy and gastroenterology specialty practice. There is a need for better characterization of this mixed IgE and non-IgE mediated food allergy, and we propose specific diagnostic criteria and an algorithm for its diagnosis. Early treatment of FPIPLE through dietary modification, would impede the ensuing atopic march. Use by clinicians of the diagnostic criteria and algorithm will avoid delay in the recognition and management of FPIPLE or even misdiagnosis. While these criteria will need modification as more information is published, at present they stand ready for future studies.

## Data Availability Statement

The original contributions presented in the study are included in the article/supplementary material, further inquiries can be directed to the corresponding author/s.

## Ethics Statement

The studies involving human participants were reviewed and approved by Clinical Hospital of Emergency for Children MS Curie, 20 Constantin Brancoveanu Avenue, 077120, Bucharest, Romania. Written informed consent to participate in this study was provided by the participants' legal guardian/next of kin. Written informed consent was obtained from the individual(s), and minor(s)' legal guardian/next of kin, for the publication of any potentially identifiable images or data included in this article.

## Author Contributions

GF and EB: conceptualization, methodology, and writing—original draft preparation. GF, AP, DI, and EB: investigation and data curation. GF, DI, and EB: resources and writing—review and editing. GF, AP, and EB: visualization. AP and EB: supervision. DI: project administration. All authors have read and agreed to the published version of the manuscript.

## Conflict of Interest

The authors declare that the research was conducted in the absence of any commercial or financial relationships that could be construed as a potential conflict of interest.

## Publisher's Note

All claims expressed in this article are solely those of the authors and do not necessarily represent those of their affiliated organizations, or those of the publisher, the editors and the reviewers. Any product that may be evaluated in this article, or claim that may be made by its manufacturer, is not guaranteed or endorsed by the publisher.

## References

[1] Boyce JA. Assa'ad A Burks AW Jones SM Sampson HA Wood RA. Guidelines for the diagnosis and management of food allergy in the united states: summary of the NIAID-sponsored expert panel report. J Allergy Clin Immunol. (2010) 126:1105–18. 10.1016/j.jaci.2010.10.007 21134568 PMC4241958

[2] Sicherer SH Warren CM Dant C Gupta RS Nadeau KC. Food allergy from infancy through adulthood. J Allergy Clin Immunol Pract. (2020) 8:1854–64. 10.1016/j.jaip.2020.02.010 32499034 PMC7899184

[3] Ho MH Wong WH Chang C. Clinical spectrum of food allergies: a comprehensive review. Clin Rev Allergy Immunol. (2014) 46:225–40. 10.1007/s12016-012-8339-6 23229594

[4] Labrosse R Graham F Caubet JC. Non-IgE-mediated gastrointestinal food allergies in children: an update. Nutrients. (2020) 12:2086. 10.3390/nu12072086 32674427 PMC7400851

[5] Burks AW Tang M Sicherer S Muraro A Eigenmann PA Ebisawa M. ICON: food allergy. J Allergy Clin Immunol. (2012) 129:906–20. 10.1016/j.jaci.2012.02.001 22365653

[6] Meyer R Fox AT Chebar Lozinsky A Michaelis LJ Shah N. Non-IgE-mediated gastrointestinal allergies—Do they have a place in a new model of the allergic March. Pediatric Allergy Immunol. (2019) 30:149–58. 10.1111/pai.13000 30403301

[7] Tracy MS Yasuda JL Rufo PA. Protein-losing enteropathy in the setting of iron deficiency anemia: a case series. JPGN Reports. (2020) 1:e009. 10.1097/PG9.0000000000000009 PMC10191603 37206595

[8] Greenberger NJ Tennenbaum JI Ruppert RD. Protein-losing enteropathy associated with gastrointestinal allergy. Am J Med. (1967) 43:777–84. 10.1016/0002-9343(67)90120-9 4168025

[9] Maloney J Nowak-Wegrzyn A. Educational clinical case series for pediatric allergy and immunology: allergic proctocolitis, food protein-induced enterocolitis syndrome and allergic eosinophilic gastroenteritis with protein-losing gastroenteropathy as manifestations of non-IgE-mediated cow's milk allergy. Pediatr Allergy Immunol. (2007) 18:360–7. 10.1111/j.1399-3038.2007.00561.x 17584315

[10] Nomura I Katsunuma T Tomikawa M Shibata A Kawahara H Ohya Y. Hypoproteinemia in severe childhood atopic dermatitis: a serious complication. Pediatric Allergy Immunol. (2002) 13:287–94. 10.1034/j.1399-3038.2002.01041.x 12390445

[11] Novembre E Leo G Cianferoni A Bernardini R Pucci N Vierucci A. Severe hypoproteinemia in infant with AD. Allergy. (2003) 58:88–9. 10.1034/j.1398-9995.2003.23710_6.x 12580819

[12] Kondo M Fukao T Omoya K Kawamoto N Aoki M Teramoto T. Protein-losing enteropathy associated with egg allergy in a 5-month-old boy. J Investig Allergol Clin Immunol. (2008) 18:63–6. 18361105

[13] Hwang JB Kang YN Won KS. Protein losing enteropathy in severe atopic dermatitis in an exclusively breast-fed infant. Pediatr Dermatol. (2009) 26:638–9. 10.1111/j.1525-1470.2009.01008.x 19840339

[14] Kitcharoensakkul M Weymann A. P342 A pediatric case of protein-losing enteropathy induced by cow's milk. Ann Allergy, Asthma Immunol. (2017) 119:S81–2. 10.1016/j.anai.2017.08.233

[15] Higuchi R Booka M Suzuki H Tsuno H. Protein-losing enteropathy and erythema caused by egg allergy in a breast-fed infant. Pediatr Int. (2016) 58:422–4. 10.1111/ped.12824 26818703

[16] Fujita Y. Nomura K. Yoshihara S. Protein-losing enteropathy in an infant with severe atopic dermatitis. BMJ Case Rep, (2021) 14:e241057. 10.1136/bcr-2020-241057 33888478 PMC8070855

[17] Braamskamp MJ Dolman KM Tabbers MM. Clinical practice. Protein-losing enteropathy in children. Eur J Pediatr. (2010) 169:1179–85. 10.1007/s00431-010-1235-2 20571826 PMC2926439

[18] Umar SB DiBaise JK. Protein-losing enteropathy: case illustrations and clinical review. Am J Gastroenterol. (2010) 105:43–9. 10.1038/ajg.2009.561 19789526

[19] Sicherer SH. Clinical aspects of gastrointestinal food allergy in childhood. Pediatrics. (2003) 111:1609–16. 10.1542/peds.111.S3.1609 12777600

[20] Mikhail I Sampson H. Eosinophilic gastrointestinal diseases. J Allergy Clin Immunol. (2016) 4:369–70. 10.1016/j.jaip.2015.07.026 26968964

[21] Dellon ES. Eosinophilic esophagitis: diagnostic tests and criteria. Curr Opin Gastroenterol. (2012) 28:382–8. 10.1097/MOG.0b013e328352b5ef 22450900 PMC4591255

[22] Steinbach EC Hernandez M Dellon ES. Eosinophilic esophagitis and the eosinophilic gastrointestinal diseases: approach to diagnosis and management. J Allergy Clin Immunol. (2018) 6:1483–95. 10.1016/j.jaip.2018.06.012 30201096 PMC6134874

[23] Robbins KA Uygungil B. Nutritional deficiencies and food allergy. J Allergy Clin Immunol. (2017) 5:528–9. 10.1016/j.jaip.2017.01.007 28283169

[24] Fahey LM Liacouras CA. Eosinophilic gastrointestinal disorders. Pediatr Clin North Am. (2017) 64:475–85. 10.1016/j.pcl.2017.01.009 28502433

[25] Kinoshita Y Furuta K Ishimaura N Ishihara S Sato S Maruyama R. Clinical characteristics of Japanese patients with eosinophilic esophagitis and eosinophilic gastroenteritis. J Gastroenterol. (2013) 48:333–9. 10.1007/s00535-012-0640-x 22847555

[26] Hill DA Grundmeier RW Ramos M Spergel JM. Eosinophilic esophagitis is a late manifestation of the allergic march. J Allergy Clin Immunol. (2018) 6:1528–33. 10.1016/j.jaip.2018.05.010 29954692 PMC6131029

